# Patients’ Satisfaction after the Treatment of Moderate Sleep Apnea and Nocturnal Bruxism with Botox or/and Thermoformed Occlusal Splints: A Pilot Study

**DOI:** 10.3390/jpm14101029

**Published:** 2024-09-26

**Authors:** Taalat Gabriel Rezk Gavrilă, Anamaria Bechir, Andrada Camelia Nicolau, Edwin Sever Bechir

**Affiliations:** 1 Doctoral School in Dental Medicine, “Titu Maiorescu” University of Bucharest, 189 Calea Vacaresti, 040056 Bucharest, Romania; rezkgavrila@gmail.com; 2 Efface Aesthetic Clinic, The CIBA Building, 146 Hagley Road, Edgbaston, Birmingham B16 9NX, UK; 3 Faculty of Dental Medicine, “Titu Maiorescu” University of Bucharest, 67A Gh. Petrascu Street, 031592 Bucharest, Romania; 4 Faculty of Medicine, Transilvania University of Brasov, 56 Nicolae Balcescu Str., 500019 Brasov, Romania; 5 Faculty of Dental Medicine, “George Emil Palade” University of Medicine, Pharmacy, Science, and Technology of Targu Mures, 38 Gh. Marinescu Street, 540142 Targu Mures, Romania; edwin.bechir@umfst.ro

**Keywords:** sleep apnea, sleep bruxism, botulinum toxin type A, occlusal splints, patient satisfaction

## Abstract

**Background**: Sleep apnea and nocturnal bruxism belong to sleep disorders that can affect the quality of life. The aim of this study was to investigate the effects on patients with moderate sleep apnea and nocturnal bruxism of Botox injection as monotherapy or associated with wearing thermoformed occlusal splints and to determine the patients’ satisfaction degree after the applied treatments. **Methods**: The selected patients for study were divided into two groups: in the first group, the patients (*n* = 18) treatment consisted of injecting Botox (Allergan) into the masseter muscle as monotherapy; in the second group, the patients (*n* = 18) benefited from associated therapy, Botox injections in masseter muscle, and the wear of thermoformed occlusal splints. At baseline, at three weeks, at three months, and six months after the effectuation of therapies, the monitoring sessions were realized. **Results**: The associated therapy presented better results in decreasing the studied symptoms than the monotherapy. Both therapies improved patient satisfaction. **Conclusions**: The applied therapies for treating the specific symptomatology in moderate sleep apnea and sleep bruxism were efficacious. Patient satisfaction was very good in both groups after the applied treatments, but the associated therapy presented better results than monotherapy.

## 1. Introduction

Sleep apnea and bruxism are sleep disorders with multifactorial etiologies, but stress is involved in their etiologies, alongside the presence of acute and chronic diseases [1].

Sleep apnea, characterized by breathing stopping when asleep, is associated with a decrease in oxygen saturation in sleep and disturbs the quality and duration of sleep, inducing the apparition of symptoms such as loud and disruptive snoring, gasping for air during sleep, and daytime fatigue [2].

The prevalence of sleep disorders has increased in recent decades. According to research realized by Lechner et al. [3], 38% of men and 30.4% of women reported snoring at night; also, 8.7% of men and 5.6% of women declared that they stopped breathing at night [4,5,6].

According to their severity, sleep apnea can be classified as mild, moderate, or severe [7,8].

The specific feature of sleep apnea is represented by recurrent pauses in respiration, which induce intermittent hypoxia, hypercapnia, and oxygen desaturation in the blood, which can suddenly increase the risk of various diseases [9].

Bruxism (teeth grinding) is a parafunctional oral activity. Teeth grinding or clenching during sleep is known as nocturnal bruxism [10,11].

Bruxism is an illness with complex pathology due to various primary or secondary factors (vicious habits such as smoking and/or alcohol/drug abuse, chronic fatigue, insomnia, occlusal imbalances, facial muscle or nerve dysfunctions, anxious states, stress, administration of antidepressants, epilepsy, etc.) [12].

The prevalence of sleep bruxism is between 22.17% and 24.89% of the European population [13]. It has been noticed that a significant factors in the apparition of sleep bruxism are age and gender (it is more frequent in women) [14].

Bruxism can be classified into nocturnal and diurnal/awake bruxism. Regarding severity, bruxism can be mild, moderate, or severe [15,16].

The characteristic symptomatology of moderate nocturnal bruxism manifests itself in the morning and is represented by facial pain, stiffness of the masticatory muscles and temporomandibular joint (TMJ), discomfort when mobilizing the temporomandibular joint, headache, and alteration of the structure of the dental hard tissues [17,18]. This condition can remain undiagnosed until the apparition of local dental lesions (repeated friction of the occlusal surface of the teeth that can induce enamel abrasion, enamel cracks, and carious lesions) and, ulteriorly, the apparition of affections in the orofacial area [19,20].

In establishing the correct diagnosis of moderate sleep apnea and moderate nocturnal bruxism, an important role belongs to the partners of the affected persons, who can observe the characteristic manifestations observed during sleep [21,22].

Sleep apnea and nocturnal bruxism negatively affect sleep quality and patient quality of life [23,24,25].

Botulinum toxin type A (BoNT-A), the purified form of the strongest poison known, is obtained from Clostridium botulinum type A. It is used for stopping the delivery of acetylcholine, and it is used for treating many diseases, like chronic sialorrhea, muscular dystonia, and spasm, but also in cosmetic applications [25,26,27,28,29].

Intraoral dental devices such as occlusal splints or thermoformed mouthpieces are recommended to decrease inappropriate occlusal forces due to nocturnal bruxism. At the same time, by increasing the space between the dental arches in occlusion, occlusal splints can be used to improve the symptoms characteristic of nocturnal apnea [30,31].

The aim of this study was to investigate the effects of Botox injection therapy, with or without wearing thermoformed occlusal splints, on patients with moderate sleep apnea and nocturnal bruxism and to determine the patients’ satisfaction degree after completing the applied treatments. The research hypothesis was that there is a correlation between the effectuated treatments (with Botox Allergan and/or occlusal splints) and the patients’ satisfaction at the end of the effectuated treatments.

## 2. Materials and Methods

This study was realized in conformity with the ethical principles and the good clinical practice of the Helsinki Declaration [32].

The procedures of this study were authorized by the Ethics Committee of the Faculty of Dental Medicine, Titu Maiorescu University in Bucharest (Decision No. 6 of 14 January 2019). This study was performed between March 2019 and April 2024, but the COVID-19 pandemic conditions caused a 14-month break in research.

### 2.1. Selection of Patients

The selected patients were properly and individually informed regarding the demands of this study, and they signed the informed consent.

All authors attended calibration courses to ensure the accuracy of each patient’s anamnesis, clinical examination, diagnosis, and then similar application of clinical procedures for the reliability of the results.

The presence of sleep apnea and nocturnal bruxism symptoms was assessed by anamnesis, oral examination, and the utilization of questionnaires for the objective and subjective symptoms and regarding the satisfaction and quality of life related to sleep bruxism and nocturnal bruxism (used at baseline and at the last monitoring session). The complaints that were taken into consideration at anamnesis of sleep apnea and nocturnal bruxism were morning headaches, facial pain or fatigue, jaw-muscle fatigue, teeth grinding or jaw clenching sounds during sleep (as observed by the family or the sleep partner), and self-perception of teeth grinding and/or jaw clenching.

The objective symptomatology of bruxism that was observed at intraoral examination was related to unphysiological tooth wear, hypertrophy or hypertonicity of the masticatory muscle bundles, trismus, tongue indentation, and buccal mucosa ridges.

The questionnaire for subjective symptoms to ascertain the patients with sleep apnea and nocturnal bruxism contained seven questions: awakening in the morning with a dry mouth or sore throat; morning pain in masseter muscles; morning fatigue; morning headaches and jaw pain; diurnal difficulty in focusing; excessive daytime sleepiness.

This questionnaire was completed by both the patients and their partners.

The selected patients (*n* = 36, 24 female and 12 male), with moderate sleep apnea and moderate nocturnal bruxism, were divided into two groups.

The first group of 18 patients (G1) was treated by injecting Botox (Allergan, AbbVie Ltd., Maidenhead, Berkshire, SL6 4UB, UK) into the masseter muscle.

The 18 patients in the second group (G2) benefited from associated therapy represented by injections with Botox and the wear of thermoformed occlusal splints applied to the mandibular dental arch.

The sample of patients participating in this study is presented in Table 1.

Inclusion criteria were the following: male and female patients between 31 and 50 years of age with good health status; moderate sleep apnea and nocturnal bruxism symptomatology; moderate tooth wear; non-smoker patients; absence of parafunctional habits; confirmed non-allergic patients for use of dental materials; the patient’s acceptance to participate in this study with signed informed consent; the possibility of patients to come for the effectuation of treatments and follow-up sessions.

Exclusion criteria were the following: Botox allergy; allergy to the materials used for thermoformed occlusal splints; more than two missing teeth in the posterior area of the dental arches; extended prosthetic restorations; adult orthodontic patients; upper respiratory and pulmonary disorders; heart disease; simulators; infections; systemic disorders/diseases; pregnancy; lactation; antipsychotic or/and psychotropic treatments; patients with mental disabilities; uncooperative patients.

**Figure 1:**
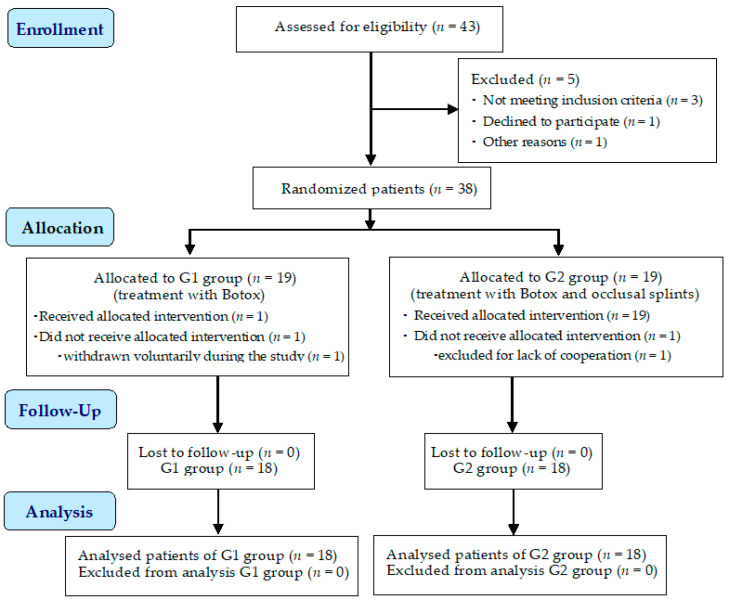
The flow chart of this study.

The flow diagram of this study is presented in Figure 1.

### 2.2. Performing Botox-Allergan Injections

Prior to beginning the therapies, a Botox allergy test was performed on each patient.

**Figure 2:**
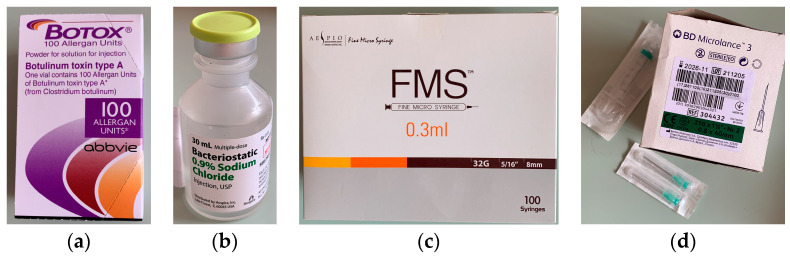
Presentation of (**a**) Botox powder; (**b**) Sodium chlorine solution; (**c**) Aespio fine micro syringe; (**d**) BD Microlance needles.

**Figure 3:**
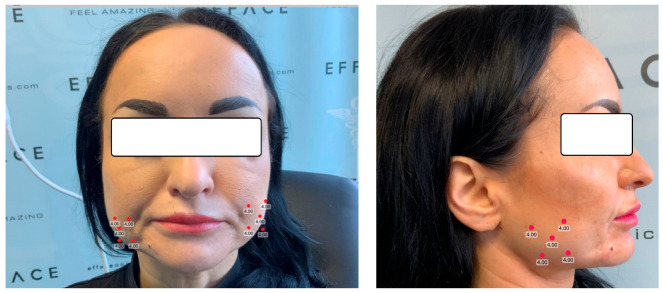
The Botox injection sites used in this study.

Botox (Allergan) powder (vacuum-dried Clostridium botulinum type A toxin) was solubilized and diluted under sterile conditions (in conformity with the manufacturer’s instructions [33,34]) with 2.5 mL of bacteriostatic 0.9% sodium chlorine per 100 units of Botox powder. Aespio fine micro syringes and BD Microlance 3 needles were used for administration of the treatment with Botox (Figure 2). The points of injection were noted after careful palpation of the temporomandibular area. In addition, measurements were made to ensure the accurate localization of each muscle bundle. A skin marker was used to map the injection points to ensure precision and reduce the risk of asymmetry. All patients were asked to clench their jaws so the masseter muscle could be identified more easily. The angle and the edge of the mandible were drawn. After measuring the drawing lines of the sides of the masseter muscle, the final draw was at least 1 cm lower than the edge of the masseter. In general, the focus was on the lower part of the masseter muscle. A total of 5 units of Botox solution were injected at 5 points on the masseters, at a distance of 1 cm between them and 1 cm from the previously drawn edges, in order to limit the possibility of affecting other muscles. The masseter muscle is a very strong muscle and needs much larger amounts of Botox than other muscles of the face. An entire ampoule of 50 units was used for the first treatment. After 3 weeks from the first treatment, the patients were recalled to verify their progress. During monitoring, after obtaining the results, if it was necessary, another 50 units of Botox were injected, but these cases were exceptions. The next assessments were after three and six months. The Botox injection sites used in this study are presented in Figure 3.

Always, bilateral Botox injections were performed, even if one side of the masseter muscle was stronger or more developed than the other. At baseline, the same amount of Botox was injected on both sides. At the first follow-up (performed after three weeks), a different quantity of Botox was injected on one side compared to the other, as needed. After these three weeks, there was a pause in the injection of Botox in order to observe how the treatment of sleep apnea and nocturnal bruxism evolves. After the last monitoring session, Botox injections were administered as needed, between one and two injections per six months, according to the faster or slower metabolism of the patients.

In order to relieve the errors and realize good investigations, objective appreciations, measurements, and treatment, all the patient’s images were captured with the “ImageJ” and “OBSERV 520” systems and preserved on “Consentz^®^ Pros” Clinic Management software system for the encrypted storage of patients’ data.

### 2.3. Manufacturing the Thermoformed Occlusal Splints

The thermoformed occlusal splints used in this study covered the mandibular dental arch in totality and were manufactured via the thermo- and vacuum processes. The hard layer of the Erkoloc-Pro sheet is made of polyethylene terephthalate glycol, and the soft layer is made of a thermoplastic polyurethane. The manufacturing was accomplished via vacuuming the softened Erkoloc-Pro sheet at approximately 130 °C on the model of hard plaster located in the Erkoform-3d+ thermoforming device (Erkodent-Erich Kopp GmbH, Pfalzgrafenweiler, Germany) [35]. After obtaining, via vacuum and pression, the specific shape of the thermoformed occlusal splints, they were processed, finished, and polished.

Thermoformed occlusal splints were distributed to the patients of the second group (G2) at baseline. These patients continued to wear the thermoformed occlusal splints even after the last assessment (six months).

### 2.4. Questionnaires Regarding the Symptomatology and the Satisfaction of Patients

Four assessments were performed (at baseline and then at three weeks, three months, and six months) to determine the presence of objective and subjective symptoms.

The objective symptoms were represented by the presence of signs of contraction in the temporal and masseter muscle bundles (hypertrophy or hypertonicity), trismus, tongue indentation, and buccal mucosa ridges.

To find out the existence of sleep apnea and nocturnal bruxism in the patients, a seven-question questionnaire was designed. These questions regarding subjective symptoms were necessary for the correct selection of patients in this study. Responses were presented in five grades: never (=strongly disagree), rarely (=disagree), sometimes (=not sure), usually (=agree), and always (=strongly agree), and were completed by the patients and by their bed partners (roommates). These questions were related to the following: awakening in the morning with a dry mouth or sore throat; morning pain in the masseter muscle; morning fatigue; morning headaches; jaw pains; diurnal (daytime) difficulty in focusing; excessive daytime sleepiness.

To reveal the satisfaction and quality of patients’ lives relating to the outcomes of applied treatments, a ten-question questionnaire was designed. Responses were presented in five grades: very dissatisfied, quite dissatisfied, neither satisfied nor dissatisfied, quite satisfied, and very satisfied. The used questions were the following: How satisfied are you now with your sleep? How satisfied are you now with your absence of contractions at bundle level in masseter muscle? How satisfied are you now with your ability to perform your daily living activities? How satisfied are you now with your capacity for work? How satisfied are you with your actual condition of concentration? How satisfied are you with the applied therapy? How satisfied are you now with your oral health? How satisfied are you now with yourself? How do you evaluate your quality of life now?

### 2.5. Statistical Analysis

The performed statistical study used the Fisher Exact Test program because the participant groups were reduced in number (G1 = 18 and G2 = 18). The Fisher Exact Test works as a possible substitute for Pearson’s chi-square test when sample sizes are small.

The Fisher Exact Test represents a good computational program for the small samples because it uses a calculator for a 2 × 2 contingency table. The test can evaluate the independence from two variables when the compared groups are not correlated.

In this pilot study, the results were studied regarding the degree of changes in patients’ life quality and in the patients’ bed partners at baseline and at the last follow-up after the treatments.

**Figure 4:**
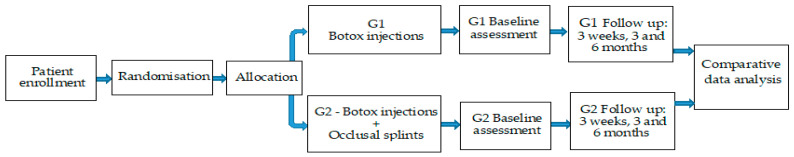
Visit schedule.

In Figure 4, the visit schedule of patients in this pilot study is depicted.

## 3. Results

During the monitoring period, at all four assessment sessions, it was noticed that all the investigated symptomatology decreased in intensity in both groups of patients (G1 and G2), which exhibited relief of discomfort in sleep apnea and nocturnal bruxism in comparison with baseline.

**Figure 5:**
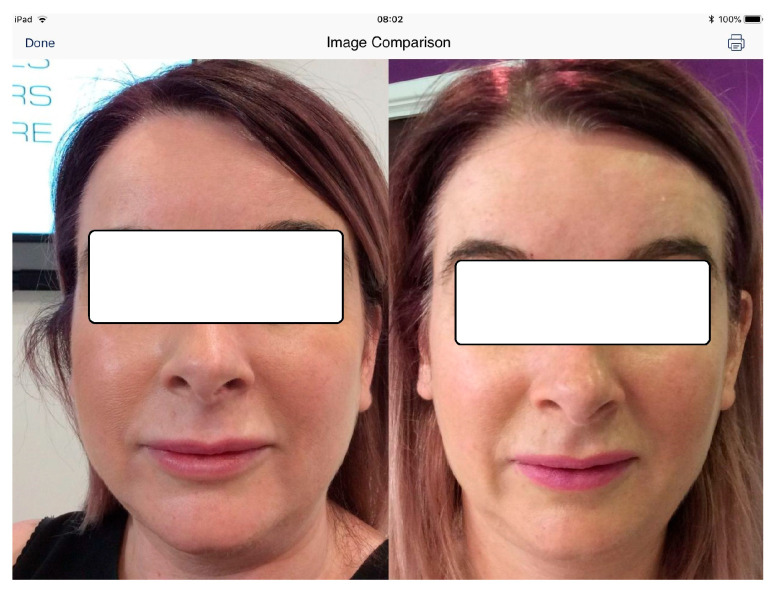
Aspect of a female patient belonging to the G2 group, at baseline (**left**) and at the fourth assessment (**right**), after the injection with Botox and wearing of thermoformed occlusal splint.

It was remarked that the therapy with Botox injections produced immediate and adequate results. It was observed that the provided results in the second group of patients (G2, which benefited from Botox-Allergan injections and wore thermoformed occlusal splints) were higher than in the first group of patients (G1, which benefited only from Botox-Allergan injections). In Figure 5, the aspect of a female patient belonging to the G2 group is presented, at baseline (left) and at the fourth assessment (right).

Table 2 presents the results regarding the studied objective symptoms, noted at baseline and at the last follow-up session.

It can be observed that initially the investigated objective symptomatology was present in all patients from both research groups (G1 and G2), and at the last evaluation session (performed at six months), no patient presented any of those objective symptoms.

The answers received from the patients and their bed partners to the questionnaire used for the subjective symptomatology of sleep apnea and nocturnal bruxism and filled out at baseline are presented in Table 3 (where never = strongly disagree; rarely = disagree; sometimes = not sure; usually = agree; always = strongly agree).

Table 4 presents the answers received at the last assessment from the patients and their bed partners to the questionnaire about the subjective symptoms of sleep apnea and nocturnal bruxism.

We mention the fact that “never” = “strongly disagree”, “rarely” = “disagree”, “sometimes” = “not sure”, “usually” = “agree”, and “always” = “strongly agree”.

It can be observed that the subjective symptomatology investigated at baseline was present in all patients from both research groups and in all patients’ bed partners. It is visible that the higher frequency was exhibited for answers with “sometimes” (not sure), “usually” (agree), and “always” (strongly agree) grades. At the last assessment (at six months), all the patients of the two investigated groups and all their bed partners had the highest frequency of subjective symptomatology noted with “never”, and the qualifiers “usually” (agree) and “always” (strongly agree) had the value 0 (=0%). The first group of patients did not wear thermoformed occlusal splints.

It should be mentioned that, at the second and third evaluation sessions (performed at three weeks and three months), all the noted values were situated between those of Table 2 and Table 4. These values were decreasing from “always” (=strongly agree) to “rarely” (disagree) and “never” (=strongly disagree) at the second and third evaluation sessions.

By studying the percentages of Table 2, Table 3 and Table 4, it can also be observed that the quality and well-being level of the life of patients were greatly improved in both groups at the end of the monitoring period compared with baseline assessment.

Table 5 presents the results regarding the changes in patients’ life quality at baseline and at the last follow-up session after applying the therapies.

The associated treatment with Botox injection and occlusal splints presented immediate results with good effects, and the patients declared that they were very satisfied with the obtained results. After the finalization of applied treatments, the filled-out questionnaires regarding the satisfaction of patients revealed that their life quality had greatly improved.

Table 6 presents the satisfaction degree in patients’ life quality after the applied treatments, noted in both groups of patients (G1: *n =* 18, only injected with Botox-Allergan, and G2: *n =* 18, who were injected with Botox and wore thermoformed occlusal splints) at baseline (before treatment) and at the fourth (last) follow-up session (after treatment).

By studying the *p*-values in Table 6 (realized with the Fisher Exact Test program), which compares the answers to the questions from the satisfaction questionnaire completed at baseline and at the end of the monitoring period, we can conclude the following:

- The surveyed patients presented the highest statistical *p* values for “sleep disruption” (*p* = 5.00), followed by “nocturnal bruxism”, “dry mouth/sore throat”, “masseter pain”, and “difficulty in focusing” with equal values (*p* < 2.00), “excessive sleepiness” with the value *p* = 2.00, and “fatigue, headaches, and jaw pain” having the lowest value *p* = 1.21;
- The surveyed patient bed partners presented the highest statistical *p* value for “waking, gasping, choking” with *p* = 5.00, followed by “mood changes” with a value of *p* = 3.32, “difficulty in focusing” with a value of *p* = 3.00, “nocturnal bruxism” with a value of *p* = 3.00, “sleep disruption” with *p* < 2.00, “excessive sleepiness” with the value of *p* = 1.29, and “snoring” with the lowest value of *p* = 1.00.

These statistical results reveal that both moderate sleep apnea and moderate sleep bruxism therapies have proven effective, and this has increased the satisfaction of all patients and their bed partners.

## 4. Discussion

Sleep apnea and bruxism are sleep disorders. These conditions can represent risk factors associated with specific clinical manifestations that affect the quality of life; therefore, it is very important that these disorders are treated [36]. Among the causes that can induce sleep disorders are depression, anxiety, constant stress, changing the time zone, various diseases (heart, stomach, kidney, or lung diseases), taking drugs that cause insomnia or drowsiness, environmental changes, aging, etc. [37].

Bruxism is an illness with complex pathology due to various primary or secondary factors (vicious habits such as smoking and/or alcohol/drug abuse, chronic fatigue, insomnia, occlusal imbalances, facial muscle or nerve dysfunctions, anxious states, stress, administration of antidepressants, epilepsy, etc.) [38,39].

The results of this study regarding therapy with Botox and occlusal splints for moderate sleep apnea and moderate sleep bruxism show that these types of treatments are non-invasive, reliable, facile to accomplish, reversible, beneficial, comfortable, and effortlessly accepted by patients.

Lobbezoo et al. [40] reported that sleep (nocturnal) bruxism is a masticatory muscle activity that occurs in sleep, and it can be distinguished as rhythmic or non-rhythmic. In healthy people, bruxism should not be considered a disorder but a behavior that may present a risk or protective factor in some particular clinical cases.

Shivamurthy et al. [41] consider that one of the predisposing factors for the development of temporomandibular disorders (which include bruxism) could be represented by stress, so screening performed at a young age could aid in the prevention and progression of the disease.

In accordance with many studies, botulinum toxin type A has proven its effectiveness in various therapies, but only when applied accurately [42,43,44,45,46].

In conformity with the review conducted by Li et al. [47], the therapy with Botox represents a reliable and efficient therapy in decreasing the pain intensity and ameliorating the functional movements of the masticatory muscles and temporomandibular joints in patients with temporomandibular dysfunctions. They consider that the dose of 60–100 U injected bilaterally can represent a beneficial solution for treating the pain that appeared due to temporomandibular dysfunctions. The rigidity of the masticatory muscles is decreased by injections with Botox, and the stiffness of the temporalis muscles is improved.

Due to the modification of masseter muscle function, changes appear in the other masticatory muscles too, which are favorable in offering functional and cosmetic outcomes. All this is achieved by the simultaneous evaluation and treatment of all the masticatory muscles, including the temporalis muscle [48].

According to Yağci et al. [49], the risk of bruxism is higher in depressed patients, those with poor quality of sleep, and those with shocking childish experiences.

The masseter muscle, although it is the strongest muscle in the human body, does not need to be weakened too much by injecting Botox, because it can lead to difficulties in the patient’s mastication, which negatively affects their functionality and quality of life [50,51]. Fortunately, any Botox treatment is completely reversible, and the muscles return to normal after a period of several months [52,53].

The beneficial mechanisms offered by the occlusal splints are not yet obvious [54], but the study conducted by Bergmann et al. [55] confirms the efficiency and safety of the use of occlusal splints in the treatment of sleep bruxism.

In their study, Albagieh et al. [56] concluded that occlusal splints can be used for the treatment of extensive variants of temporomandibular disorders (TMDs), as well as bruxism, headaches, reduced vertical dimension of occlusion, and so on, but there is no evident proof that treatment with occlusal splints is better than physiotherapy in the treatment of TMDs.

After the review realized by Duarte et al. [57], at the moment, there is not adequate scientific evidence to advocate or confirm the existence of a link between sleep, bruxism, and quality of life in the general population.

In their study, Mercan Başpınar et al. [25] observed that decreased oral health in relation to the quality of life and low sleep quality would be expected in the presence of nocturnal bruxism. They underlined the fact that the patients may not be aware of their situation until it is clearly presented by a dentist.

Because the etiology of sleep apnea and sleep bruxism is multifactorial, interdisciplinary collaboration is necessary to increase the success rate of treatment, and the ideal treatment options are represented by the multidisciplinary approaches [58,59].

The systematic review effectuated by Ferrillo [60] indicates that a multidisciplinary approach involving rehabilitation dentistry and physical medicine as well as psychology is compulsory for the suitable diagnosis and treatment of patients with sleep disorders.

Regarding the effectuated statistical data, initially, the simple Chi-test program was applied, but, unfortunately, the results were not relevant at all, and the values obtained were noted as “erroneous”. For this reason, the performed statistical study was with the Fisher Exact Test program. The *p* values obtained after applying this program confirmed the fact that the results of this study were relevant.

Despite the small sample sizes, this pilot study can contribute to the progress of clinical knowledge, and also, through the clinical and statistical significance, it has the possibility to contribute to the further development of clinical practice guidelines.

The clinical relevance of this pilot study is given by the favorable results obtained in the therapy with Botox with or without thermoformed occlusal splints, for moderate sleep apnea and moderate sleep bruxism. The limitations of this pilot study are due to the limited number of patients in the study groups and the relatively short time interval of the monitoring.

## 5. Conclusions

Associated therapy with Botox with or without thermoformed occlusal splints of moderate sleep apnea and moderate sleep bruxism represented a reliable and efficacious treatment for decreasing the specific symptomatology of these afflictions.

Also, the results of this study demonstrated that the applied therapies improved patient satisfaction and their quality of life.

Sleep apnea and sleep bruxism have significant implications for the health status and life satisfaction of those affected, so these conditions require a personalized approach to both diagnosis and management. Future studies with a larger number of patients with moderate sleep apnea and bruxism are needed.

In addition, the study of the cumulative action of factors such as gender, ages, smoking, consumption of alcohol, caffeine, drugs, and addictive substances can complete the interpretation of studies and of working hypotheses.

## Figures and Tables

**Table 1:** Sample of the patients participating in this study.

	All Patients	Group 1 (G1)	Group 2 (G2)
No of patients	36	18	18
Age (mean ± years)	31–50 year	Male	33–50 (mean 41.5 ± 8.5)
		Female	31–48 (mean 39.5 ± 8.5)
Gender M/F	Male 12 (34%)	6 (33.33%)	6 (33.33%)
	Female 24 (66%)	12 (66.66%)	12 (66.66%)

**Table 2:** The results regarding the studied objective symptoms, noted at baseline and at the last follow-up session.

	Objective Symptom	Patients Group	Baseline		Last Follow-Up Session	
			Present	Absent	Present	Absent
1.	Contraction of the temporal muscle bundles	G1	18 (100%)	0 (0%)	0 (0%)	18 (100%)
		G2	18 (100%)	0 (0%)	0 (0%)	18 (100%)
2.	Contraction of the masseter muscle bundles	G1	18 (100%)	0 (0%)	0 (0%)	18 (100%)
		G2	18 (100%)	0 (0%)	0 (0%)	18 (100%)
3.	Trismus	G1	18 (100%)	0 (0%)	0 (0%)	18 (100%)
		G2	18 (100%)	0 (0%)	0 (0%)	18 (100%)
4.	Tongue indentation	G1	18 (100%)	0 (0%)	0 (0%)	18 (100%)
		G2	18 (100%)	0 (0%)	0 (0%)	18 (100%)
5.	Buccal mucosa ridges	G1	18 (100%)	0 (0%)	0 (0%)	18 (100%)
		G2	18 (100%)	0 (0%)	0 (0%)	18 (100%)

**Table 3:** Questionnaire for the patients and their bed partners about subjective symptoms: responses at baseline.

Questions Addressed to Patients (*n* = 36)		Responses	Questions Addressed to Patients’ Bed Partners (*n* = 36)		Responses
Sleep disruption (episodes of stopping breathing)	never	3 (=8.33%)	Sleep disruption (episodes of stopping breathing)	never	0 (=0%)
	rarely	11 (=30.55%)		rarely	1 (=2.77%)
	sometimes	5 (=13.88%)		sometimes	2 (=5.55%)
	usually	11 (=30.55%)		usually	4 (=11.11%)
	always	6 (=16.66%)		always	29 (=80.55%)
Nocturnal bruxism (teeth grinding or clenching)	never	0 (=0%)	Nocturnal bruxism (teeth grinding or clenching)	never	1 (=2.77%)
	rarely	1 (=2.77%)		rarely	3 (=8.33%)
	sometimes	6 (=16.66%)		sometimes	6 (=16.66%)
	usually	9 (=25.0%)		usually	12 (=33.33%)
	always	20 (=55.55%)		always	14 (=38.88%)
Awakening in the morning with a dry mouth or sore throat	never	0 (=0%)	Snoring	never	0 (=0%)
	rarely	4 (=11.11%)		rarely	2 (=5.55%)
	sometimes	7 (=19.44%)		sometimes	13 (=36.11%)
	usually	12 (=33.33%)		usually	12 (=33.33%)
	always	13 (=36.11%)		always	9 (=25.0%)
Morning pain in the masseter muscle	never	0 (=0%)	Waking during the night and gasping or choking	never	0 (=0%)
	rarely	3 (=8.33%)		rarely	4 (=11.11%)
	sometimes	4 (=11.11%)		sometimes	5 (=13.88%)
	usually	9 (=25.0%)		usually	19 (=52.77%)
	always	20 (=55.55%)		always	8 (=22.22%)
Morning fatigue, headaches, and jaw pain	never	0 (=0%)	Mood changes (e.g., depressive or irritable)	never	3 (=8.33%)
	rarely	5 (=13.88%)		rarely	9 (=25.0%)
	sometimes	8 (=22.22%)		sometimes	8 (=22.22%)
	usually	12 (=33.33%)		usually	9 (=25.0%)
	always	11 (=30.55%)		always	7 (=19.44%)
Diurnal (daytime) difficulty in focusing	never	0 (=0%)	Diurnal (daytime) difficulty in focusing	never	1 (=2.77%)
	rarely	1 (=2.77%)		rarely	3 (=8.33%)
	sometimes	12 (=33.33%)		sometimes	5 (=13.88%)
	usually	12 (=33.33%)		usually	16 (=44.44%)
	always	11 (=30.55%)		always	12 (=33.33%)
Excessive daytime sleepiness	never	1 (=2.77%)	Excessive daytime sleepiness	never	6 (=16.66%)
	rarely	8 (=22.22%)		rarely	6 (=16.66%)
	sometimes	9 (=25.0%)		sometimes	7 (=19.44%)
	usually	7 (=19.44%)		usually	8 (=22.22%)
	always	11 (=30.55%)		always	9 (=25.0%)

**Table 4:** Questionnaire for the patients and their bed partners about subjective symptoms: responses at the last follow-up session.

Questions Addressed to Patients (*n* = 36)		Responses	Questions Addressed to Patients’ Bed Partners (*n* = 36)		Responses
Sleep disruption (episodes of stopping breathing)	never	30 (=83.33%)	Sleep disruption (episodes of stopping breathing)	never	26 (=72.22%)
	rarely	2 (=5.55%)		rarely	6 (=16.66%)
	sometimes	3 (=8.33%)		sometimes	4 (=11.11%)
	usually	1 (=2.77%)		usually	0 (=0%)
	always	0 (=0%)		always	0 (=0%)
Nocturnal bruxism (teeth grinding or clenching)	never	31 (=86.11%)	Nocturnal bruxism (teeth grinding or clenching)	never	27 (=75%)
	rarely	3 (=8.33%)		rarely	6 (=16.66%)
	sometimes	1 (=2.77%)		sometimes	3 (=8.33%)
	usually	1 (=2.77%)		usually	0 (=0%)
	always	0 (=0%)		always	0 (=0%)
Awakening in the morning with a dry mouth or sore throat	never	31 (=86.11%)	Snoring	never	30 (=83.33%)
	rarely	4 (=11.11%)		rarely	3 (=8.33%)
	sometimes	1 (=2.77%)		sometimes	2 (=5.55%)
	usually	0 (=0%)		usually	1 (=2.77%)
	always	0 (=0%)		always	0 (=0%)
Morning pain in the masseter muscle	never	32 (=88.88%)	Waking during the night and gasping or choking	never	28 (=77.77%)
	rarely	2 (=5.55%)		rarely	5 (=13.88%)
	sometimes	2 (=5.55%)		sometimes	3 (=8.33%)
	usually	0 (=0%)		usually	0 (=0%)
	always	0 (=0%)		always	0 (=0%)
Morning fatigue, headaches, and jaw pain	never	29 (=80.55%)	Mood changes (e.g., depressive or irritable)	never	30 (=83.33%)
	rarely	4 (=11.11%)		rarely	3 (=8.33%)
	sometimes	3 (=8.33%)		sometimes	3 (=8.33%)
	usually	0 (=0%)		usually	0 (=0%)
	always	0 (=0%)		always	0 (=0%)
Diurnal (daytime) difficulty in focusing	never	26 (=72.22%)	Diurnal (daytime) difficulty in focusing	never	27 (=75%)
	rarely	7 (=19.44%)		rarely	7 (=19.44%)
	sometimes	3 (=8.33%)		sometimes	2 (=5.55%)
	usually	0 (=0%)		usually	0 (=0%)
	always	0 (=0%)		always	0 (=0%)
Excessive daytime sleepiness	never	28 (=77.77%)	Excessive daytime sleepiness	never	29 (=80.55%)
	rarely	4 (=11.11%)		rarely	4 (=11.11%)
	sometimes	4 (=11.11%)		sometimes	3 (=8.33%)
	usually	0 (=0%)		usually	0 (=0%)
	always	0 (=0%)		always	0 (=0%)

**Table 5:** Results regarding the degree of changes in patients’ life quality after the applied treatments (baseline and last follow-up).

	Group	Very Dissatisfied		Quite Dissatisfied		Neither Satisfied Nor Dissatisfied		Quite Satisfied		Very Satisfied	
		B	LFu	B	LFu	B	LFu	B	LFu	B	LFu
How satisfied are you with your sleep?	G1	12 66.66%	0 0%	2 11.11%	0 0%	2 11.11%	0 0%	1 5.55%	2 11.11%	1 5.55%	16 88.88%
	G2	11 61.11%	0 0%	3 16.66%	0 0%	2 11.11%	0 0%	1 5.55%	1 5.55%	1 5.55%	17 94.44%
How satisfied are you about the contractions in masseter muscle?	G1	10 55.55%	0 0%	4 22.22%	0 0%	2 11.11%	1 5.55%	1 5.55%	2 11.11%	1 5.55%	15 83.33%
	G2	12 66.66%	0 0%	3 16.66%	0 0%	2 11.11%	0 0%	1 5.55%	1 5.55%	0 0%	17 94.44%
How satisfied are you now about your ability to perform daily living activities?	G1	11 61.11%	0 0%	5 27.77%	0 0%	1 5.55%	0 0%	1 5.55%	2 11.11%	0 0%	16 88.88%
	G2	12 66.66%	0 0%	4 22.22%	0 0%	1 5.55%	0 0%	1 5.55%	1 5.55%	0 0%	17 94.44%
How satisfied are you now with your capacity for work?	G1	10 55.55%	0 0%	5 27.77%	0 0%	2 11.11%	0 0%	1 5.55%	2 11.11%	0 0%	16 88.88%
	G2	10 55.55%	0 0%	4 22.22%	0 0%	2 11.11%	0 0%	2 11.11%	1 5.55%	0 0%	17 94.44%
How satisfied are you now with your condition of concentration?	G1	12 66.66%	0 0%	3 16.66%	0 0%	1 5.55%	1 5.55%	1 5.55%	2 11.11%	1 5.55%	15 83.33%
	G2	13 72.22%	0 0%	2 11.11%	0 0%	1 5.55%	0 0%	1 5.55%	1 5.55%	1 5.55%	17 94.44%
How satisfied are you now with the applied therapy?	G1	0 0%	0 0%	0 0%	0 0%	0 0%	0 0%	0 0%	1 5.55%	0 0%	17 94.44%
	G2	0 0%	0 0%	0 0%	0 0%	0 0%	0 0%	0 0%	0 0%	0 0%	18 100%
How was the nocturnal comfort with your occlusal splint?	-	-	-	-	-	-	-	-	-	-	-
	G2	3 16.66%	0 0%	3 16.66%	0 0%	3 16.66%	1 5.55%	4 22.22%	2 11.11%	5 27.77%	15 83.33%
How satisfied are you now with the health of your orofacial system?	G1	6 33.33%	0 0%	6 33.33%	0 0%	4 22.22%	0 0%	3 16.66%	2 11.11%	1 5.55%	16 88.88%
	G2	6 33.33%	0 0%	6 33.33%	0 0%	3 16.66%	0 0%	3 16.66%	1 5.55%	0 0%	17 94.44%
How satisfied are you now with yourself?	G1	6 33.33%	0 0%	5 27.77%	0 0%	3 16.66%	1 5.55%	3 16.66%	3 16.66%	1 5.55%	14 77.77%
	G2	6 33.33%	0 0%	4 22.22%	0 0%	4 22.22%	0 0%	3 16.66%	2 11.11%	1 5.55%	16 88.88%
How do you evaluate now your overall quality of life?	G1	3 16.66%	0 0%	5 27.77%	0 0%	4 22.22%	0 0%	3 16.66%	2 11.11%	3 16.66%	16 88.88%
	G2	4 22.22%	0 0%	3 16.66%	0 0%	4 22.22%	0 0%	4 22.22%	1 5.55%	3 16.66%	17 94.44%

**Table 6:** Statistical results regarding the degree of changes in patients’ life quality after the applied treatments (at baseline and last follow-up session).

		Baseline					Last Follow-Up Session					*p*-Value *p* (Chi sqr)
		Never	Rarely	Sometime	Usual	Always	Never	Rarely	Sometime	Usual	Always	
Patients	Sleep disruption	3	11	5	11	6	30	2	3	1	0	=5.00
	Nocturnal bruxism	0	1	6	9	20	31	3	1	1	0	<2.00
	Dry mouth/sore throat	0	4	7	12	13	31	4	1	0	0	<2.00
	Masseter pain	0	3	4	9	20	32	2	2	0	0	<2.00
	Fatigue, headaches, jaw pain	0	5	8	12	11	29	4	3	0	0	=1.00
	Difficulty in focusing	0	1	12	12	11	26	7	3	0	0	<2.00
	Excessive sleepiness	1	8	9	7	11	28	4	4	0	0	=2.00
Patient bed partners	Sleep disruption	0	1	2	4	29	26	6	4	0	0	<2.00
	Nocturnal bruxism	1	3	6	12	14	27	6	3	0	0	=3.00
	Snoring	0	2	13	12	9	30	3	2	1	0	=1.00
	Waking, gasping, choking	0	4	5	19	8	28	5	3	0	0	=5.00
	Mood changes	3	9	8	9	7	30	3	3	0	0	=3.00
	Difficulty in focusing	1	3	5	16	12	27	7	2	0	0	=3.00
	Excessive sleepiness	6	6	7	8	9	29	4	3	0	0	=1.00

## Data Availability

Data are contained within the article.
